# Dynamic microglial activation is associated with LPS-induced depressive-like behavior in mice: An [18F] DPA-714 PET imaging study

**DOI:** 10.17305/bjbms.2021.6825

**Published:** 2022-01-30

**Authors:** Tian Qiu, Jiamei Guo, Lixia Wang, Lei Shi, Ming Ai, Zhu Xia, Zhiping Peng, Li Kuang

**Affiliations:** 1 Department of Psychiatry, The First Affiliated Hospital of Chongqing Medical University, Chongqing, China; 2 Department of Nuclear Medicine, The First Affiliated Hospital of Chongqing Medical University, Chongqing, China; 3 Department of Radiological Medicine, College of Basic Medicine, Chongqing Medical University, Chongqing, China

**Keywords:** Depression, microglia, [18F] DPA-714, lipopolysaccharide, positron emission tomography

## Abstract

Major depressive disorder is a highly pervasive, severe psychological condition for which the precise underlying pathophysiology is incompletely understood, although microglial activation is known to play a role in this context. Here, we analyzed the association between neuroinflammation and depressive-like behaviors in a lipopolysaccharide (LPS)-induced mouse model system using 10–12-week-old male C57BL/6 mice. Microglial activation and associated neuroinflammatory activity were monitored through positron emission tomography (PET) imaging. Animals were assessed at 3 time points, including 24 hours prior to LPS injection, 24 hours post-LPS injection, and 72 hours post-LPS injection. Analyses of microglial activation and hippocampal neuroinflammation were conducted through [18]F DPA-714 PET imaging and immunohistochemical staining for ionized calcium-binding adapter molecule 1 and translocator protein. Moreover, NOD-like receptor protein 3 (NLRP3) inflammasome activity and interleukin-1β (IL-1β) levels were assessed at 24 hours post-LPS injection. We found that the LPS treatment was associated with a marked increase in depressive-like behavior at 24 hours post-injection that was less pronounced at the 72 hours post-injection time point. These changes coincided with enhanced [18F] DPA-714 PET uptake in the whole brain, hippocampus, cortex, and amygdala together with increased hippocampal microglial activation as evidenced by immunofluorescent staining. By 72 hours post-injection, however, these PET and immunofluorescence phenotypes had returned to baseline levels. Furthermore, increased NLRP3 inflammasome activation and IL-1β expression were evident at 24 hours post-LPS injection. These data demonstrate that dynamic microglial activation is associated with LPS-induced depressive-like behaviors and hippocampal neuroinflammation in a mouse model system.

## INTRODUCTION

The World Health Organization (WHO) estimates that over 264 million individuals worldwide suffer from major depressive disorder (MDD) [1], which is forecast to impose one of the greatest disease-related economic burdens by 2030 [2]. Current treatments for depression are not satisfactory, with sustained remission and appropriate disease management only being achieved in half of patients, while 20% of patients fail to respond at all to therapeutic intervention [3]. There is, thus, a clear unmet need for the development of novel treatments for this condition. The pathogenesis of MDD has been shown to be closely related to neuroinflammation [4], with psychological disorders having been linked to infectious or autoimmune disease-related inflammatory processes in some contexts [5,6]. This systemic inflammation can be recapitulated in rodent model systems through the peripheral injection of lipopolysaccharide (LPS) or other immunostimulatory compounds [7]. Systemic LPS administration can thus be used to stimulate glial cells and to induce the generation of pro-inflammatory cytokines including interleukin-1β (IL-1β), which likely plays an important role in these pathological processes given that IL-1β receptor knockdown has been shown to alleviate rodent neuropsychiatric behavioral dysfunction [8]. The NOD-like receptor family comprising 3 (NLRP3) inflammasome directly controls IL-1β maturation and release, highlighting this signaling mechanism as a key link between stress and the activation of inflammatory immune processes involved in the development of MDD [9].

Microglia serve as key regulators of neuroinflammation within the central nervous system (CNS), in addition to performing other key homeostatic functions including synaptic maintenance, the removal of cellular debris, pathogen surveillance, and trophic support. The activation of microglia is a common finding in patients with depression that has been tied to neuronal apoptosis and inflammatory cytokine production [10]. Positron emission tomography (PET) using the second-generation translocator protein (TSPO) ligand [18F] DPA-714 can directly assess microglial activation *in vivo*, offering a robust and effective means of monitoring neuroinflammatory activity within the CNS in a sensitive manner in the context of neurological disease [11,12]. Relative to other radiotracers, [18F] DPA-714 exhibits an extended isotope half-life, reduced non-specific binding, higher affinity, and superior bioavailability within the brain [13-15]. However, efforts to apply PET imaging as a tool when studying MDD have been relatively limited to date, and there have been only limited reports employing *in vivo* imaging to assess LPS-induced rodent models of depression.

Herein, we implemented a multimodal longitudinal approach to monitor the mechanisms governing MDD-related behaviors in an LPS-induced mouse model system. Dynamic changes in neuroinflammation over time in these mice were monitored through microglial PET-CT scanning and analyses of microglial markers, NLRP3, and cytokine expression. We also evaluated both depressive behavior and microglia-related processes in an effort to clarify the correlations between these two sets of variables.

## MATERIALS AND METHODS

### LPS-induced MDD mouse model establishment

**FIGURE 1:**
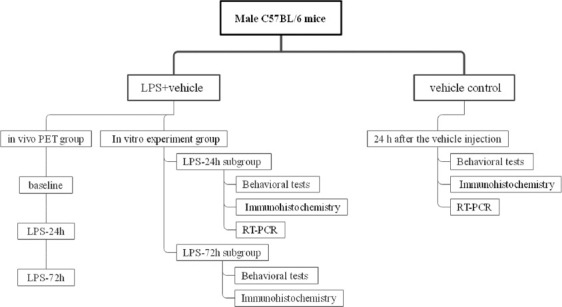
LPS-induced MDD mouse model establishment protocol.

C57BL/6 mice (10–12 weeks old, 18–25 g) were obtained from Chongqing Medical University (Chongqing, China). Mice were housed (2–4 mice/cage) with free access to water and food in a facility with a 12 hours regular light/dark cycle (8:00 AM on–8:00 PM off). The same animals were housed in cages over time. Following a 2-week acclimatization period during which mice were randomized into a vehicle control group (n = 25) and an LPS (*Escherichia coli*, serotype 055:B5, Sigma-Aldrich Chemical, St. Louis, USA) model group (n = 70). Mice in the model group were subdivided into an LPS-24 hours group and an LPS-72 hours mouse group. LPS model mice were intraperitoneally (i.p.) injected with LPS (1 mg/kg in PBS), while vehicle controls were injected with an equivalent volume of PBS (10 mL/kg). At 24 hours post-injection, control mice and those in the LPS-24 hours group underwent behavioral testing including the tail suspension test (TST), open field test (OFT), and sucrose preference test (SPT). Then, 8–10 mice per group were euthanized to collect brain tissue samples (Figure 1). At 72 hours post-injection, the LPS-72 hours mice were then subjected to behavioral testing, followed by the collection of brain tissue samples for immunofluorescent staining. PET-CT analyses were conducted using nine male mice that were scanned at 24 hours before LPS injection (baseline), 24 hours post-LPS injection (LPS-24 hours), and 72 hours post-LPS injection (LPS-72 hours).

### Microglia PET imaging

#### PET radiotracer

The [18F] DPA-714 radiotracer was obtained from the Nuclear Medicine Department of The First Affiliated Hospital of Chongqing Medical University. It was modified with a commercially available Sumitomo Heavy Industries CFN 100 Pro synthesizer based on previously published protocols, yielding an 18–36% solution of ready-to-inject, > 99% radiochemically pure [18F]DPA-714 (formulated in physiological saline containing 10% ethanol) [13].

#### Nano-PET-CT analyses

[18F] DPA-714 PET imaging was conducted at baseline and at the indicated time points (24 and 72 hours post-LPS injection). For imaging, mice were intravenously (i.v.) injected with ~ 8.0 MBq of [18F] DPA-714 in 200 mL of PBS through the tail vein. Mice were then permitted to stay in their home cages for 40 minutes during radiotracer uptake. Before PET scanning, mice were anesthetized using isoflurane (4% and 2% in oxygen for induction and maintenance, respectively). A single, 20 minutes static PET scan was then initiated, after which CT scans (500 µA, 80 kV, 98 µm, and 360º rotation in 220 steps) were conducted. [18F] DPA-714 PET scans were conducted using a nanoScan PET/CT (Mediso Ltd., Budapest, Hungary) platform at the selected experimental time points, with murine respiration and body temperature being monitored throughout the scanning process with an M2M-BioVet™ small animal physiological monitoring system (M2M imaging, OH, USA). After scanning was complete, mice were returned to the home cages and allowed to awaken. The OSEM 2D algorithm was employed for list-mode data reconstruction (frames, 4 × 30, 5 × 150, 6 × 450 s) without correcting for attenuation. Reconstructed PET assessments were manually aligned with the murine brain MRI template atlas using PMOD v3.7 (PMOD Technologies, Zurich, Switzerland; www.pmod.com). The cortex, hippocampus, and amygdala were selected as volumes of interest (VOIs). Regional brain uptake values for all mice were given as standardized uptake values (SUVs) and recorded for 0–20 minutes after PET scanning. SUVs were calculated for the 0–20-minute time frame as follows:


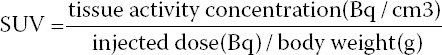


### Behavioral testing

Behavioral testing was performed for mice in the control and LPS groups between 8:00 AM and 12:00 PM, with real-time data being captured on video for subsequent analysis using an automated animal behavior recording analysis and software platform (SMART Video-Tracking System; Panlab, Spain). Testing was conducted in a quiet environment (background noise <65 dB).

#### OFT

OFT was employed as in prior reports to assess spontaneous anxiety- and exploration-related behaviors [16]. Briefly, the bottom of an aluminum alloy box (50 cm × 50 cm × 50 cm) was subdivided into 25 equally sized square regions, with those squares that were and were not in contact with the wall being, respectively, referred to as the outer perimeter and the central region (The 9 squares in the middle). Mice were separately placed into the center of this grid and monitored for 6 minutes, with the final 5 minutes of this period being recorded for the scoring of distance traveled and the number of rearing behaviors. A video tracking system was utilized to analyze the recorded video as a means of calculating both the total time spent and distance traveled in the central zone of the open field.

#### TST

Despair-related behavior was assessed via a TST approach based on a modified version of a published protocol [17]. Briefly, mice were suspended 30 cm above the group with a tiny piece of tape placed 2 cm from the tip of the tail. After being allowed to acclimate to these conditions for 2 minutes, mice were recorded on video. These videos were then analyzed using the SMART software with a preset tracking frame and a threshold for motion speed. Depressive-like reactions consistent with behavioral despair were assessed based on the immobility time (s) during which mice were hanging without struggling.

#### SPT

Depressive-like anhedonic behaviors in mice were assessed via the SPT based on a published protocol [18]. During acclimatization, mice were trained to consume a 1% sucrose solution by housing them in cages containing a single bottle of 1% sucrose during the first 24 h, followed by housing with one bottle containing water and one containing 1% sucrose during the following 24 h. The positions of these bottles did not remain consistent in the testing cages. During the experimental testing phase, sucrose preferences for mice in each group were assessed based on the changes in weight of the bottles containing water and 1% sucrose. The ratio of sucrose intake to total water intake was used to quantify sucrose preference.

### Brain tissue preparation

At appropriate time points, three mice per group were selected at random and euthanized through the i.p. injection of 1% pentobarbital sodium (0.4 ml/100 g), followed by fixation achieved through perfusion with 4% paraformaldehyde (2 ml/kg body weight). Brain tissue was then harvested and split into two hemispheres along the midsaggital line, with the right or light hemisphere being selected at random and cut into 3–5 um sections with a cryostat microtome (CM1860, Leica). Every ten slices were selected at random from among sections containing the hippocampal region, with six sections on average per series, yielding ten sampling profile groups.

#### qPCR

The chloromethane reagent (Servicebio, China) was used to prepare total RNA from hippocampal tissue samples, followed by the use of a First Strand cDNA Synthesis Kit (Servicebio, China) to prepare cDNA. SYBR Green qPCR Master Mix (High ROX) (Servicebio, China) was then used to conduct qPCR analyses using the following primers: IL-1β (F 5′ GCATCCAGCTTCAAATCTCGC 3′; R 5′TGTTCATCTCGGAGCCTGTAGTG 3′), NLRP3 (F 5′TAAGAACTGTCATAGGGTCAAAACG 3′; R5′GTCTGGAAGAACAGGCAACATG 3′), ASC (F 5′ACTATCTGGAGTCGTATGGCTTGG 3′; R 5′ TTCTGTGACCCTGGCAATGAG 3′), caspase-1 (F 5′GGCTGACAAGATCCTGAGGG 3′; R 5′TAGGTCCCGTGCCTTGTCC 3′), TSPO (F 5′GCAGAAACCCTCTTGGCATC 3′; R 5′ AGCGTCCTCTGTGAAACCTCC 3′). Relative mRNA expression was assessed through the 2^−DDCt^ method, with GAPDH being used for normalization.

#### Immunofluorescent staining

Ionized calcium-binding adaptor molecule-1 (Iba-1) and glial fibrillary acidic protein (GFAP) are considered to be markers for microglia and reactive astrocytes, respectively. Mice were sacrificed immediately after behavioral testing. Immunofluorescence assays were performed as described previously. Briefly, mice were deeply anesthetized with pentobarbital sodium and transcranially perfused with 4% paraformaldehyde in 0.01 M phosphate buffer. The brain tissue was removed and fixed in 4% paraformaldehyde and stored at 4°C for immunofluorescence analysis. The brains were dehydrated, paraffin-embedded, and cut into 3–5 μm sections. After being deparaffinized, the sections were heated in a microwave oven for 25 minutes in EDTA antigen retrieval buffer (pH 8). Subsequently, the tissue sections were immersed in 3% H_2_O_2_ and incubated in goat serum in 3% BSA (Servicebio, Wuhan, China) for 30 minutes. The tissue sections were then incubated at 4°C overnight with a mixture of anti-TSPO (1:2000, Abcam, UK) and anti-Iba-1 (1:1000, Novus, USA) or anti-GFAP antibodies (1:2000, Abcam, UK). After washing with PBS, sections were incubated with fluorescein isothiocyanate (FITC)-conjugated goat anti-rabbit IgG (1:100, Servicebio, Wuhan, China) and tetramethylrhodamine isothiocyanate (TRITC)-conjugated goat anti-mouse IgG (1:100, Servicebio, Wuhan, China) for 90 minutes at 37°C. The sections were then washed with PBS and incubated with a DAPI solution for 10 minutes. Then, the sections were mounted on slides and cover slipped. Fluorescence was assessed via laser scanning confocal microscopy (Leica Microsystems Heidelberg GmbH, Germany) with an Olympus SP2 inverted microscope (Olympus) equipped with a Fluoview FVX confocal scan head. Hippocampal subregions were identified based on DAPI staining, and sections were oriented accordingly. The number of Iba-1+, TSPO+, TSPO+/Iba-1+, GFAP+, and TSPO+/GFAP+ cells was evaluated. The morphology of microglia was studied using Image J (1.42q).

### Ethical statement

This study was carried out in accordance with the National Institutes of Health Guide for the Care and Use of Laboratory Animals. The protocol was approved by the Animal Ethics Committee of The First Affiliated Hospital of Chongqing Medical University, Chongqing, China (2021-772).

### Statistical analysis

GraphPad Prism 8.4.3 was used to analyze data, which are given as the mean ± standard error of the mean (SEM). The D’Agostino and Pearson test was used to evaluate whether data conformed to a normal distribution, while the Brown-Forsythe test was used to evaluate the homogeneity of variance. One-way ANOVAs followed by Tukey’s *post hoc* test were used to compare data if the variance was similar; otherwise, the Brown-Forsythe test was applied, and the Dunnett’s T3 multiple comparisons test was employed for further comparisons. *p* < 0.05 was the threshold of significance.

## RESULTS

### LPS exposure induces depressive-like behavior in mice

**FIGURE 2:**
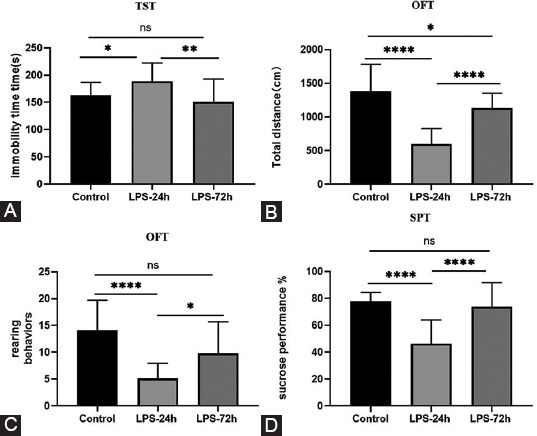
LPS induces depressive-like behavior in mice. (A) Time spent immobile in the TST; (B) Total distance traveled in the OFT; (C) Rearing behaviors in the OFT; (D) Sucrose performance. All data are expressed as the mean ± SEM. One-way ANOVA was used for *p < 0.05, **p < 0.005, ****p < 0.0001, compared to each group.

Consistent with prior reports, C57BL/6 mice injected with LPS (1 mg/kg) developed depressive- and anxiety-like behaviors at 24 hours post-injection relative to control animals. As shown in Figure 2, observed LPS injection-induced depressive behaviors at this time point included an enhanced immobility duration and diminished struggling time in the TST (*P* = 0.0128), reduced mobility, decreased total distance traveled, and fewer rearing behaviors in the OFT (*p* < 0.0001), and decreased sucrose consumption in the SPT (*p* < 0.0001). However, these effects were largely reversed at 72 hours post-LPS injection, with immobility time in the TST being significantly reduced as compared to that in LPS-injected mice at the 24 hours time point to a level not different from that in controls (*P* = 0.523) and a significant increase in sucrose consumption relative to that observed at the 24 hours time point that was not substantially distinct from that observed in control animals (*P* = 0.736). OFT analyses conducted in mice at 72 hours post-injection revealed increased total distance traveled, and more rearing behaviors, as compared to those observed in LPS-injected mice at the 24 hours time point (*p* < 0.0001). However, relative to the normal control group, the total distance traveled by LPS-72 mice was still reduced (*P =* 0.028).

### [F18]-DPA-714 PET-CT imaging analyses

**FIGURE 3:**
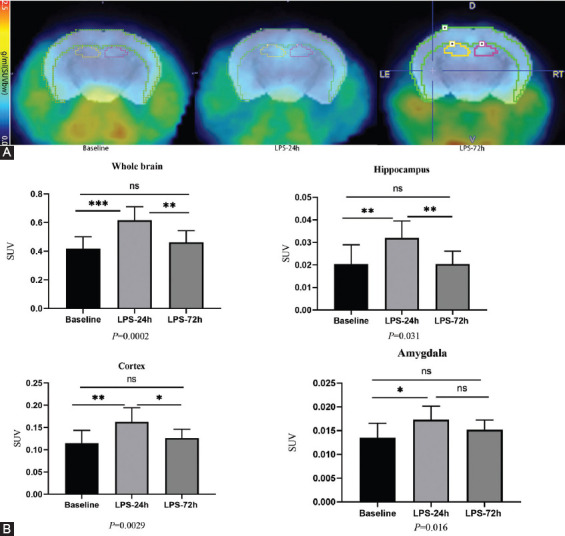
*In vivo* PET imaging can detect lps-induce neuroinflammation. 9 Mice were i.v. injected with [18F]DPA-7 14 at 24 hours before (baseline), and 24 hours and 72 hours post IP injection of LPS to quantitatively assess brain neuroinflammation in terms of Regional brain uptake values were given as standardized uptake values (SUV). (A) The PET images were aligned with the mouse brain MRI template atlas. We selected the hippocampus, cortex and amygdala as the VOIs and calculated their SUV. PET/MRJ template atlas fusion images; (B) DPA-714 accumulation (n = 9/group). We selected the Whole brain, Hippocampus, Cortex, and Amygdala as the VOIs. All data are expressed as the mean ± SEM. The results of the one-way ANOVA were *p < 0.05,**p < 0.005, ***p < 0.001, compared to baseline group.

Next, we employed a [18F] DPA-714 Nano-PET imaging approach to monitor levels of TSPO in LPS-injected mice over time (baseline, 24 hours post-injection, and 72 hours post-injection) as a tool to assess microglial activation. SUV values (g/mL) were used to monitor [18F] DPA-714 uptake in the whole brain and in specific regions of interest including the cortex, hippocampus, and amygdala (Figure 3). Quantitative analyses revealed significant increases in [18F] DPA-714 signal in the whole brain, the cortex, and hippocampus at 24 hours post-LPS injection that had returned to baseline at 72 hours post-injection, consistent with substantial but transient microglial activation. Baseline and 24 hours post-injection SUVs were as follows: cortex (0.11 ± 0.010 vs. 0.16 ± 0.011 g/ml, *P* = 0.0029), hippocampus (0.020 ± 0.003 vs. 0.032 ± 0.003 g/ml, *P* = 0.008), whole brain (0.42 ± 0.028 vs. 0.62 ± 0.032 g/ml, *P* = 0.0002), and amygdala (0.0135 ± 0.003 vs. 0.0173 ±0.002 g/ml, *P* = 0.015). SUVs at 24 hours and 72 hours post-LPS injection were as follows: cortex (0.16 ± 0.011 vs. 0.13 ± 0.007 g/ml, *P* = 0.025), hippocampus (0.032 ± 0.003 vs. 0.020 ± 0.002 g/ml, *P* = 0.007), whole brain (0.62 ± 0.032 vs. 0.46 ± 0.024 g/ml, *P* = 0.0027), and amygdala (0.0173 ± 0.002 vs. 0.0152 ±0.002 g/ml, *P* = 0.384). There were no substantial differences between LPS-72h and baseline SUVs, consistent with microglial activation having returned to baseline: cortex (0.13 ± 0.007 vs. 0.11 ± 0.010 g/ml, *P* = 0.64), hippocampus (0.020 ± 0.002 vs. 0.020 ± 0.003 g/ml, *P* = 0.99), whole brain (0.46 ± 0.027 vs. 0.42 ± 0.028 g/ml, *P* = 0.53), and amygdala (0.0152 ±0.002 vs. 0.0135 ± 0.003 g/ml, *P* = 0.232).

### LPS exposure increases the expression of microglia markers and alters microglial morphology

**FIGURE 4:**
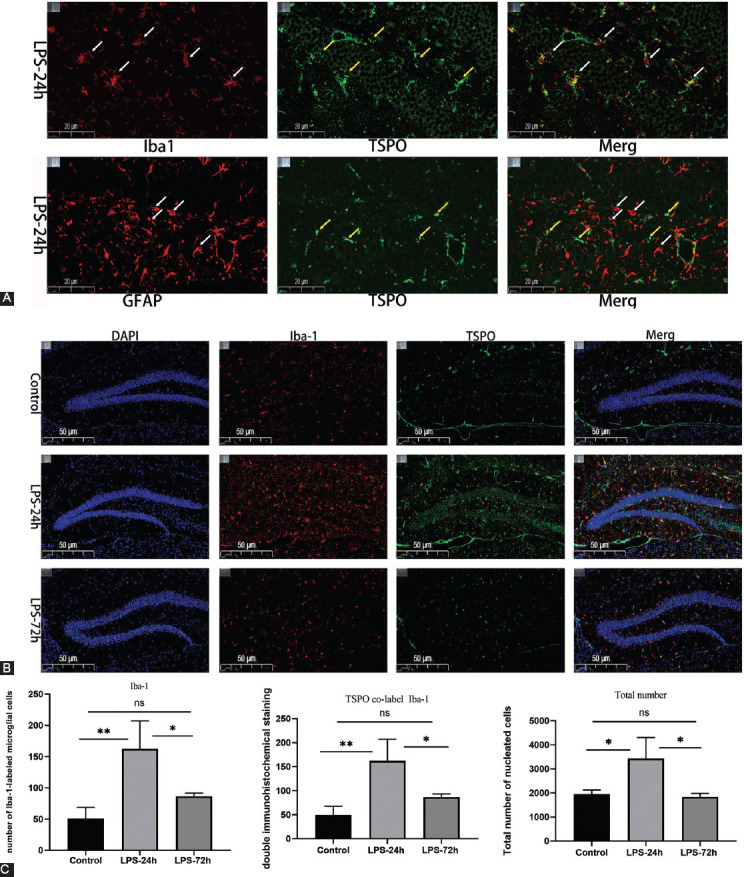
LPS exposure increased microglial marker expression. (A) Immunofluorescence staining of hippocampal sections from LPS-24 hours mice. TSPO (green), Iba-I\GAPF (red); scale bar, 20 μm. Arrows indicate the cells positive for Iba-I and TSPO. LPS injection induces microglia activation, and TSPO is predominantly expressed on Iba-I labeled microglia; (B) Iba-1/TSPO/DAPI (red/green/blue) staining in the hippocampus; scale bar, 50 mm; (C) Qualification ofiba-1 immunofluorescence density (n = 5/group). All data are expressed as the mean ± SEM, analysis by one-way ANOVA, *p < 0.05, **p < 0.005.

Next, immunofluorescent staining of brain tissue samples from control and LPS-injected mice (24 hours and 72 hours) was performed to evaluate correlations between [18F] DPA714-PET signals, activated microglia, and TSPO levels. A dual-staining approach was used to detect the expression of TSPO and Iba-1 or GFAP within the hippocampal region to determine whether TSPO was primarily expressed by microglia (Iba-1+) or reactive glial cells (GFAP+). As shown in Figure 4A, LPS injection-induced significant microglial activation, with TSPO being primarily expressed on Iba-1+ microglia rather than on GFAP+ astrocytes. These data further confirmed a strong correlation between [18F] DPA-714 signal and microglial activation. Visual analysis of immunofluorescent TSPO, Iba-1, and DAPI-stained tissue sections from mice in these three different treatment groups revealed that specimens collected at 24 hours post-LPS injection exhibited the highest levels of microglial activation (Figure 4B). Relative to control samples, those from the LPS-24h group exhibited more activated microglia and significantly elevated TSPO levels. However, no differences in TSPO levels or microglial activation were evident when comparing the control and LPS-72 hours samples, consistent with a return to baseline phenotypes. As shown in Figure 4C, quantitative analyses revealed that relative to control brain sections, samples from mice collected at 24 hours post-LPS injection exhibited increased Iba-1+ activated microglia (*p* < 0.005), a higher number of TSPO+ Iba-1+ cells (*p* < 0.005), and a rise in the total number of cells in the hippocampus (*p* < 0.05). Relative to LPS-24 hours mice, LPS-72 hours mice exhibited a reduction in the number of activated microglia in the hippocampus (*p* < 0.05), with fewer cells exhibiting dual TSPO and Iba-1 positivity (*p* < 0.05) and a reduction in total cell number (*p* < 0.05). No differences in these variables were observed when comparing samples from control and LPS-72 hours mice.

**FIGURE 5:**
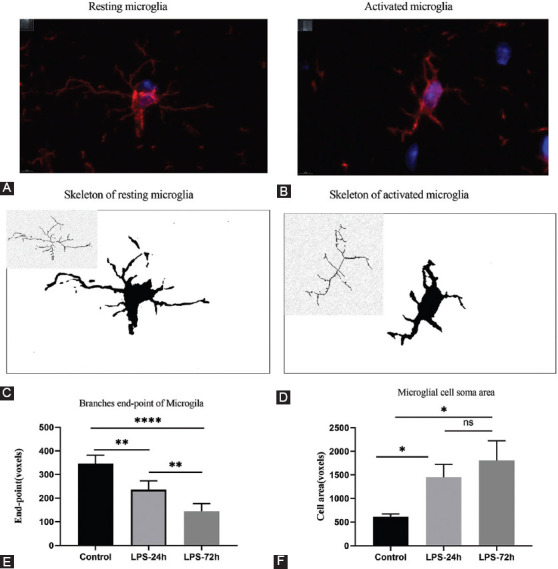
The morphological study of microglia analyzed by Image J. (A) Resting microglia under immunofluorescence; (B) Activated microglia under immunofluorescence; (C) Resting microglia cytoskeleton; (D) Activated microglia cytoskeleton; (E) Control, LPS-24 hours and LPS-72 hours hippocampal microglia branch endpoint analysis by one way ANOVA analysis, *p < 0.05, **p < 0.005, ****p < 0.0001. All data are expressed as the mean ± SEM. (F) Control, LPS-24 hours and LPS-72 hours hippocampal microglial cell soma area analysis by one-way ANOVA analysis, *p < 0.05. All data are expressed as the mean ± SEM.

The morphology of microglia can be classified descriptively, or it can be quantified based upon continuous parameter variables including cell branching, complexity, and shape. The AnalyzeSkeleton (2D/3D) plugins were used to collect morphological data from fluorescent and bright field images converted into representative binary and skeletonized images in Image J [19]. The outputs of these plugins summarized cell morphology in terms of process endpoints, as well as cell shape and size descriptors. As shown in Figure 5A and C, the resting microglia in the control group were branched with smaller cell bodies and longer processes. After LPS stimulation, microglia were activated, exhibiting an amoeboid shape with an enlarged cell body and shortened processes (Figure 5B and D). The hippocampal tissue sections from different groups were repeatedly delineated the region of interest (ROI) for morphological analyses of these microglia via one-way ANOVAs, Branch endpoint analysis results suggested that there were significant reductions in these endpoints in the LPS-24 hours and LPS-72 hours groups relative to the control group (*p* < 0.0001) (Figure 5E). In a comparative study of cell area (Figure 5F), the average area of cell soma in the control group was 640 voxels, which was significantly smaller than that observed in the LPS-24 hours group (1448 voxels) and the LPS-72 hours group (1809 voxels) (*p* < 0.05). Both these single-cell description studies and multicellular quantitative analyses of the hippocampal region revealed that microglia were activated after LPS injection.

### LPS injection induces hippocampal IL-1β and NLRP3 inflammasome upregulation

**FIGURE 6:**
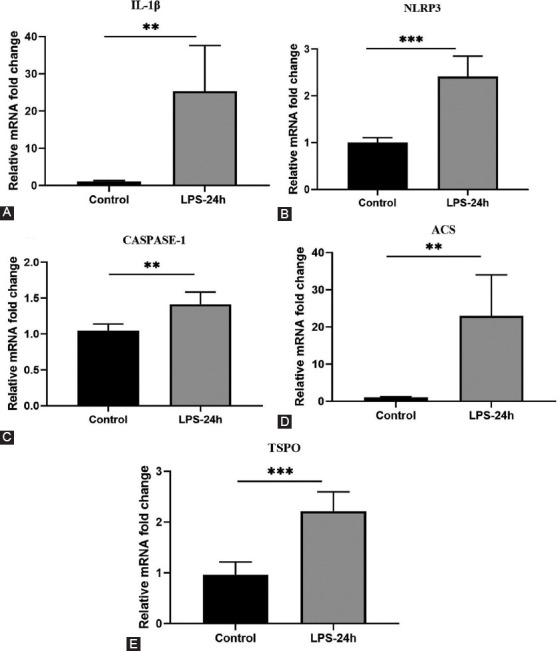
Detection of cytokine changes in the hippocampus of LPS-24 hours mice. (A-E) Real-time PCR analysis of IL-1β, NLRP3, CASPASE-l, ASC, TSPO (n = 5/group). All data are expressed as the mean ± SEM. *t*-test used by*p < 0.05, **p < 0.005, compared to normal control.

To more fully evaluate the effects of LPS injection on the hippocampus in this murine model system, we evaluated the expression of inflammation-related genes at 24 hours post-LPS injection. At the mRNA level, hippocampal IL-1β expression was significantly increased at 24 hours post-LPS injection relative to the levels observed in control mice (Figure 6A, *P* = 0.0022). The NLRP3 inflammasome and downstream caspase-1 cleavage are essential for IL-1β maturation, leading us to further evaluate the expression of key components of this signaling pathway in our experimental mice. Hippocampal mRNA levels of the NLRP3 inflammasome components NLRP3 (Figure 6B, *P* = 0.0001), Caspase-1 (Figure 6C, *P* = 0.0028), ASC (Figure 6D *P* = 0.0022), and TSPO (Figure 6E, *P* = 0.0003) were markedly elevated at 24 hours post-LPS injection relative to those in control mice, consistent with a role for the NLRP3 inflammasome as a regulator of LPS-induced depressive-like behavior.

## DISCUSSION

In this study, we conducted a comprehensive longitudinal analysis of neuroinflammation in an LPS-induced murine model of depression through 18[F] DPA-714 PET imaging. Our PET imaging data and *ex vivo* Iba-1 and TSPO staining of brain tissue sections enable us to conclude that activated microglia are cellular correlates associated with enhanced [18F] DPA-714 uptake in this model system. We employed [18F] DPA-714 PET to observe microglial activation-related neuroinflammation and increased TSPO levels in the hippocampus and cortex at 24 hours post-LPS injection, while we found that such inflammation had largely returned to baseline by 72 hours post-injection. This evidence of short-term inflammation, together with the observed upregulation of hippocampal NLRP3 and IL-1β mRNA levels, may be indicative of a robust transient innate immune response to LPS exposure. Overall, these findings suggest that neuroinflammation contributes to LPS-induced depression symptoms, with [18F] DPA-714 PET imaging offering a means of monitoring dynamic microglial activation in this murine model system.

Current animal models fail to recapitulate the complexity of MDD. However, LPS-based model systems still offer value as a tool for antidepressant screening and assessing the pathogenesis of this disease [20]. A single systemic LPS injection is sufficient to induce depressive-like behaviors consistent with acute systemic infection or inflammation [21]. In line with prior reports, we found that our mice demonstrated depressive-like behaviors at 24 hours post-LPS injection as evidenced by reduced consumption of sucrose in the SPT, enhanced immobility and reduced struggling in the TST, and a reduction in total distance traveled in the OFT. We also detected elevated pro-inflammatory cytokine expression and higher levels of microglial activation in the LPS-24 hours mice, consistent with neuroinflammation observed in prior histological studies [22]. Low-dose LPS challenge has also been reported to activate microglia within 6 hours, with such activation persisting for at least 72 hours [23]. However, we found depressive-like behavioral symptoms to be most apparent at 24 hours post-LPS injection whereas they had largely returned to baseline at the 72 hours time point, with these results coinciding with the timing of maximal microglial activation, suggesting a correlation between depressive-like behaviors at the activation of these cells.

Many studies have been conducted to date exploring a range of preclinical imaging modalities, demonstrating the value of small-animal PET scanning in the context of neuroinflammation as a robust and quantitative approach to longitudinal noninvasive *in vivo* imaging. Researchers have previously employed TSPO PET imaging time series to characterize neuroinflammation associated with animal disease models of conditions including Alzheimer’s disease [24], stroke [25], seizure [26], and multiple sclerosis [27]. Despite much progress in this research space, the precise role of microglial activation in the context of MDD dynamics remains poorly understood, and there have been no previously published studies to our knowledge evaluating the use of *in vivo* TSPO imaging as a tool for monitoring inflammation in the brain in the context of LPS-induced depression. Using repeated PET scanning of the same mice over time, we determined that the [18F] DPA-714 signal in the whole brain, hippocampus, cortex, and amygdala rose significantly at 24 hours following LPS injection before falling to baseline levels at 72 hours post-injection. These data are consistent with prior evidence that intraperitoneally injected LPS can induce diffuse neuritis in the brain [23], with inflammation subsequently becoming concentrated in regions tied to depression including the cerebral cortex and hippocampus. These regions may be linked to the onset of depressive symptoms in humans [28]. The observed temporospatial neuroinflammation-related TSPO-PET imaging data generated herein coincide well with data from prior IHC-based analyses of inflammation-induced and stress-related depression models [29]. Moreover, these data align with recent evidence regarding increased TSPO levels in the brains of MDD patients relative to healthy age-matched controls as detected through [18F] FEPPA PET imaging [30]. Microglial activation levels have been reported to be increased in individuals suffering from advanced depression who had not taken antidepressants for an extended period as compared to patients that had only not taken these antidepressants for a short time interval, consistent with the activation of these microglia being a gradual process rather than a static aspect of the underlying pathology [31]. The specific degree to which these activated astrocytes contribute to TSPO signal increases has yet to be firmly established [32]. However, recent evidence suggests that activated microglia are the most important contributor to such expression. We additionally found that IHC staining for Iba-1 was a reliable approach to activated microglia detection within the hippocampus. By conducting dual Iba-1/TSPO staining, we were thereby able to confirm the localization of TSPO to microglia within the murine hippocampus. Our data show that peripherally-induced neuroinflammation can be reliably detected in the CNS using the TSPO-PET radioactive tracer [18F] DPA-714. Our results supported the conclusion that the resulting increase in [18F] DPA-714 TSPO signaling is primarily attributable to a combination of microglia, astrocytes, and monocyte-derived macrophages [33]. This study focused on the expression of the DPA-714 signal in glial cells. One limitation of this study is that we were not able to further distinguish the source of cellular signals in PET imaging. In the future, novel tracers are expected to overcome this limitation. In this study, we found that after multiple PET scans, there were dynamic changes in the uptake values of the whole brain and some brain regions, such as the cortex and amygdala. At present, we only conducted *in vitro* validation of our *in vitro* experiments by assessing hippocampal section. In the future, additional brain regions will be assessed through *in vitro* experiments based on the possible changes in the brain regions in this PET study.

Both neuroinflammation and systemic inflammatory activity are closely linked to the pathogenesis of mental health disorders and neurodegenerative disease [34,35]. Short-term neuroinflammatory activity can be tied to immunological activities including the removal of pathogens or cellular debris, contributing to neuronal death and the exacerbation of neurodegenerative diseases [35]. The precise innate immune mechanisms that are engaged within the CNS, however, remain unclear.

In this study, we observed altered microglial morphology at 24 and 72 hours post-LPS injection, with activated microglia exhibiting enlarged cell bodies, shorter processes, and a rounded or rod-like shape. Activated microglia further exhibited the loss of cellular processes, amoeboid morphology, and phagocytic functionality. LPS-activated M1 microglia secrete pro-inflammatory factors [23]. Herein, we assessed the hippocampal expression of the pro-inflammatory cytokine IL-1β, revealing it to be markedly upregulated at 24 hours post-LPS injection relative to baseline. Many bacteria, viruses, and endogenous stress-related stimuli can induce the NLRP3 inflammasome-mediated activation of caspase-1, which, in turn, cleaves the precursor form of IL-1β to produce a mature form thereof which can participate in ongoing inflammatory processes [36]. Three different NLRP3 inflammasome genes (NLRP3, ASC, and Caspase-1) were found to be substantially upregulated at the mRNA level following LPS treatment relative to baseline, consistent with a central role for this pathway in LPS-induced depression.

## CONCLUSION

Given the consistency with histological findings, these data underscore the potential value of *in vivo* TSPO PET imaging as a reliable means of assessing depression-related encephalitis. The detailed longitudinal analysis of the processes of microglial activation will serve as a foundation for future research regarding the optimal timing of anti-inflammatory treatments for MDD. Additional longitudinal imaging-based studies have the potential to offer more robust evidence confirming the utility of TSPO imaging as a prognostic biomarker associated with the development of depression.
